# Supplementary material for: Genome-wide association studies unveil major genetic loci driving insecticide resistance in Anopheles funestus in four eco-geographical settings across Cameroon
Source: BMC Genomics. 2024 Dec 18;25:1202. doi: 10.1186/s12864-024-11148-7 (PMC11654272; doi:10.1186/s12864-024-11148-7)
Supplement: Supplementary file 1 — Supplementary Material 1 [file 12864_2024_11148_MOESM1_ESM.pdf]

# Supplementary information

## Supplementary Figures

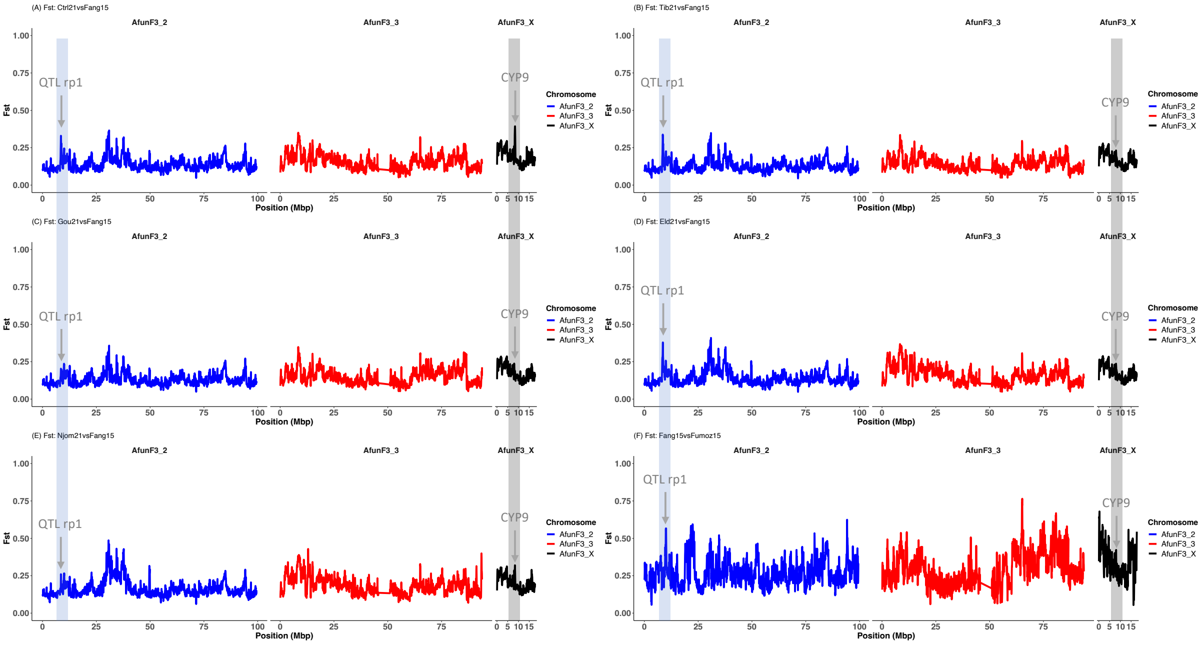


**S1 Fig.** Pairwise $F_{ST}$ genetic differentiation between *Anopheles funestus* population from four eco-geographical settings and FANG.

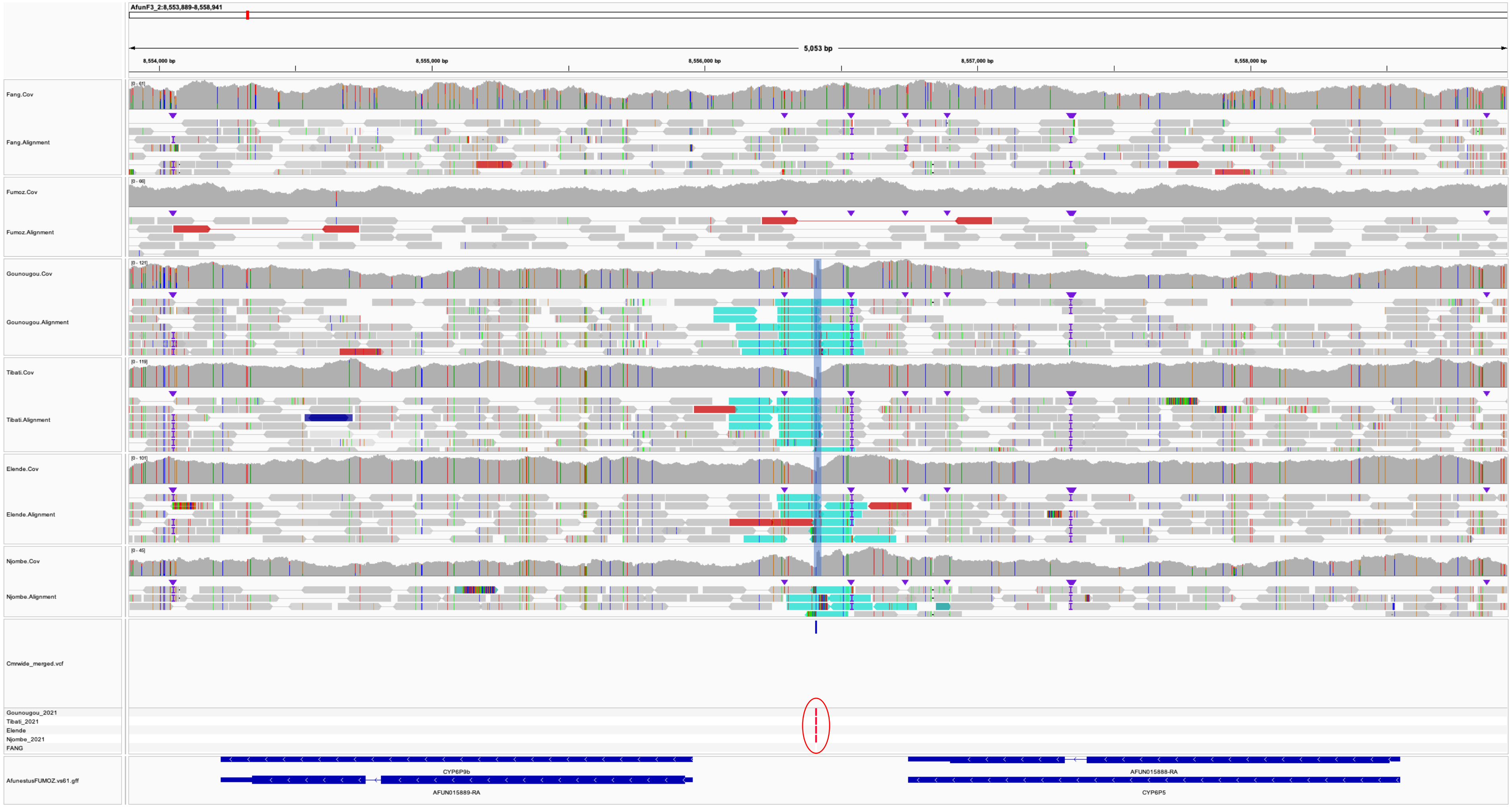


**S2 Fig.** Evidence of a 4.3kb transposon insertion between CYP6P9b and CYP6P5 is observed in *Anopheles funestus* populations across Cameroon. The screenshot from Integrative Genomics Viewer (IGV) displays coverage depth and aligned reads for pooled template whole genome sequences of FANG, FUMOZ, Gounougou, Tibati, Elende, and Njombe. The red markers in the circle indicate the TE insertion, displayed with a characteristic pattern in blue vertical rectangle. The coverage depth plots reveal increased coverage downstream of the TE insertion in all populations. The grey rectangles separated by thin lines represent normal reads aligned in pairs, while green rectangles represent discordant reads.

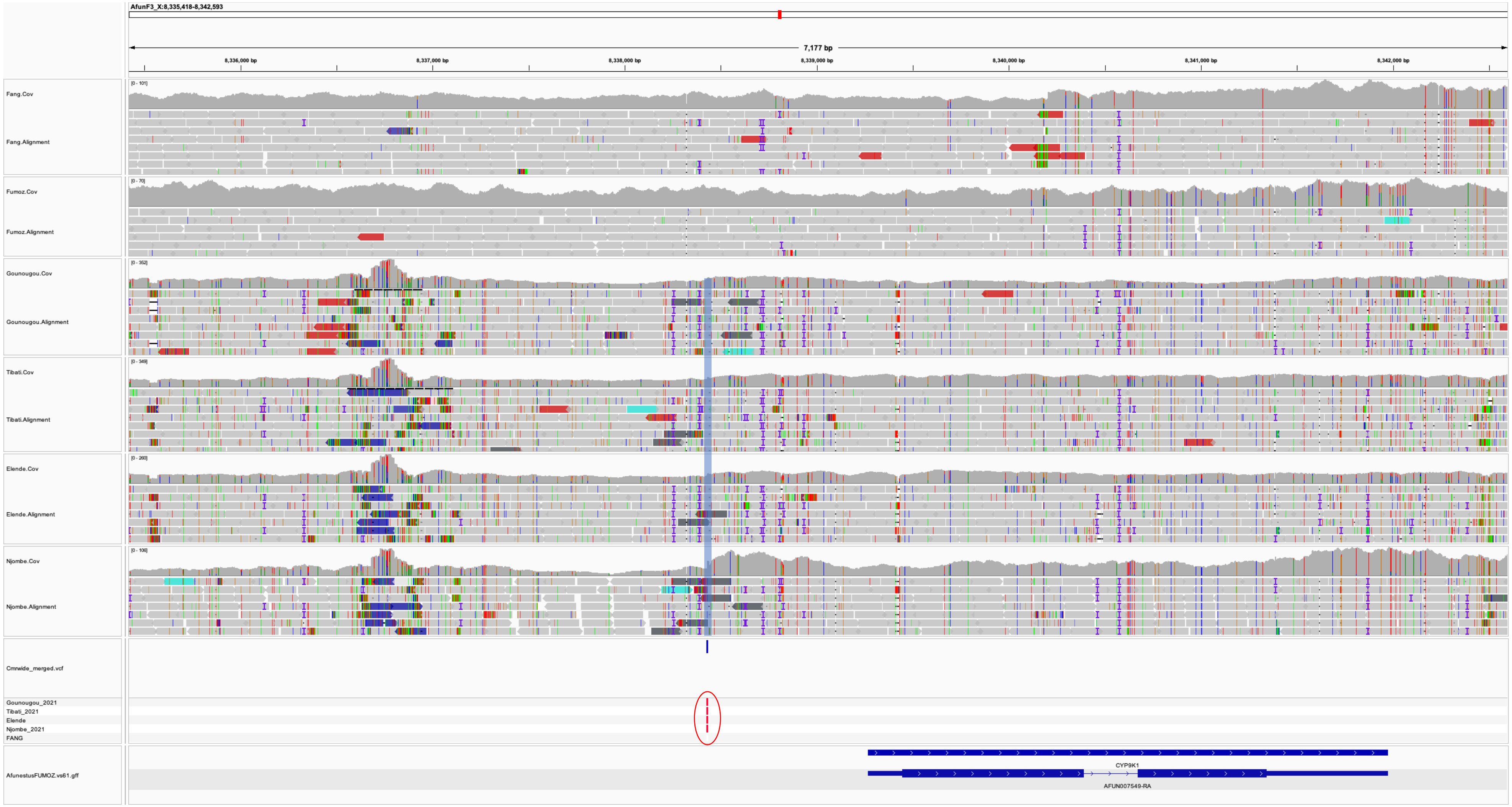


**S3 Fig.** Evidence of a substantial transposon insertion, with an unknown size, is observed upstream of the CYP9K1 gene in *Anopheles funestus* populations across Cameroon. The red markers in circle indicate TE insertion in all populations, characterized by a pattern as blue vertical rectangle, apparently more prevalent in Njombe population compared to others. The coverage depth plots reveal increased coverage downstream of the TE insertion in the Njombe population but not in others, including FANG and FUMOZ. The grey rectangles represent normal reads aligned in pairs, while thick black rectangles represent discordant reads.

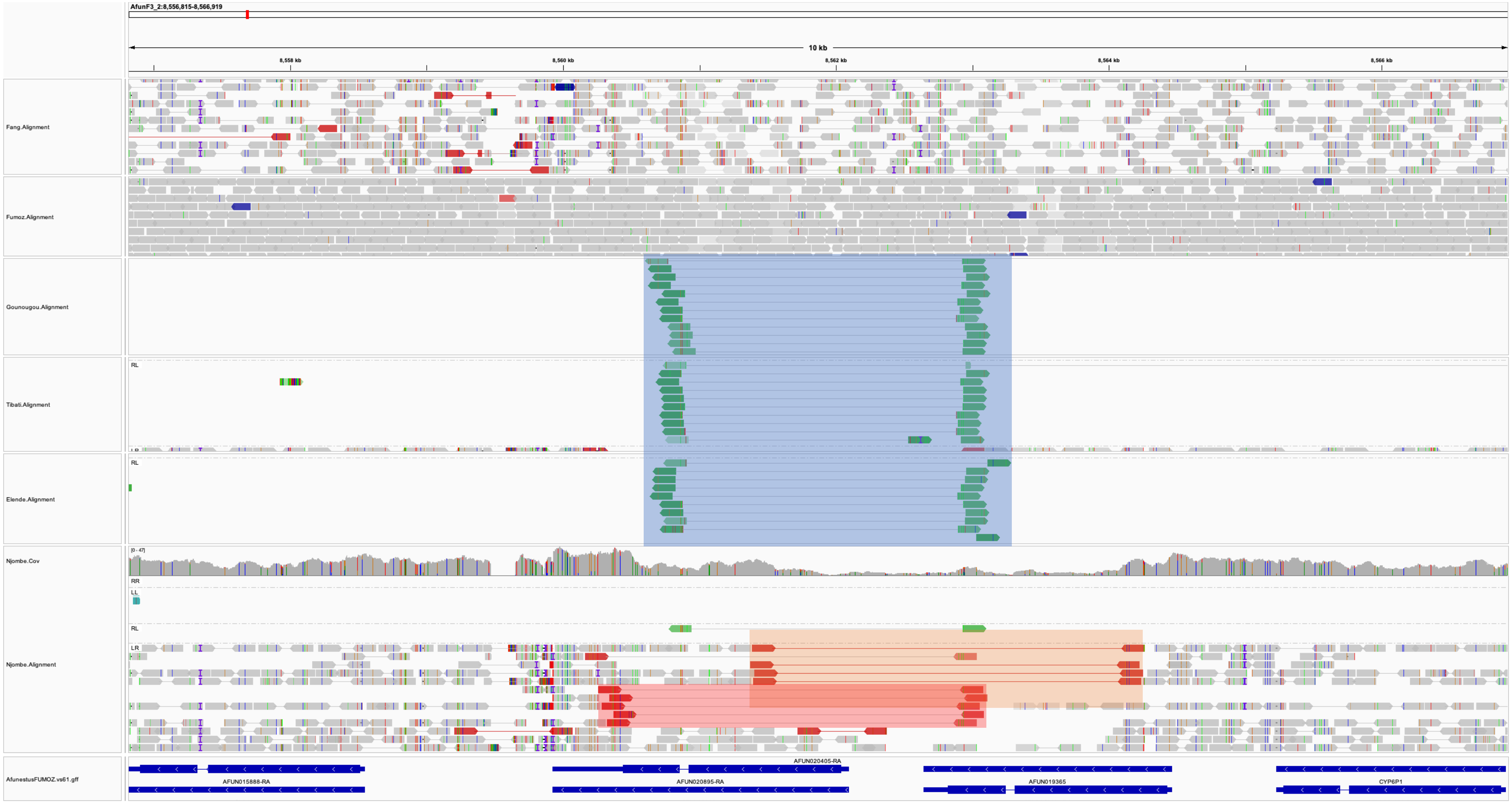


**S4 Fig.** Evidence of a 2.4kb duplication, spanning partial CYP6P4a and b paralogues, is observed with an unusual insert size, represented by green rectangles separated with thin lines and framed in blue boxe. These duplications are present in Gounougou, Tibati, and Elende but absent in Njombe, FANG, and FUMOZ. A red framed box with reads of unusual sizes, separated by red thin lines, corresponds to a 2.3kb deletion found in all populations, while the orange framed box represents a 2.5kb deletion only found in the Njombe population. The drop in coverage is noticeable in the Njombe population coverage track. Grey rectangles, separated by thin lines, represent normal aligned reads.

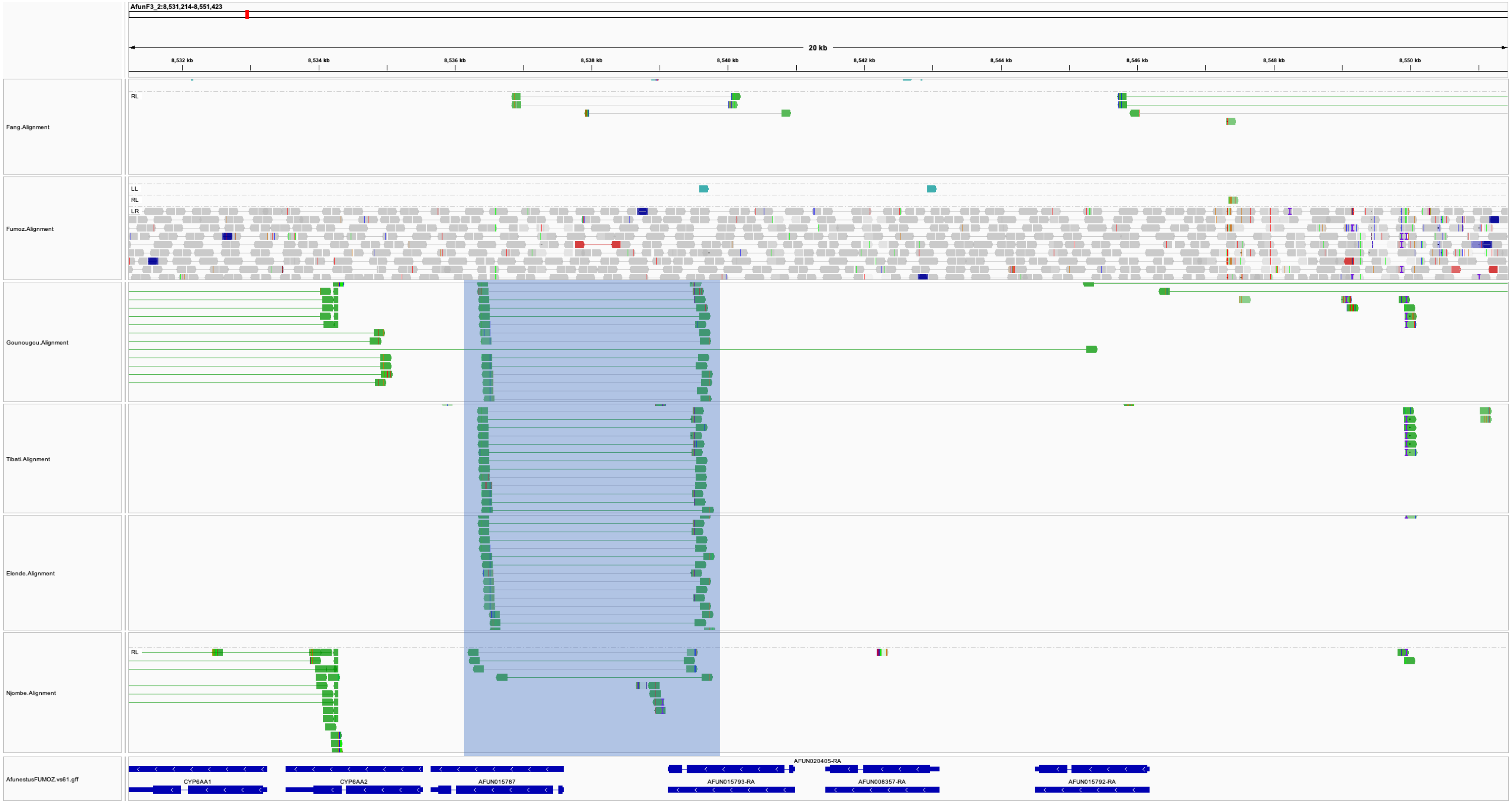

**S5 Fig.** Evidence of a 3.5kb duplication, spanning partial 2x carboxylesterases, is represented by an unusual insert size as green rectangles, separated by a thin line and framed in a blue box. This duplication is consistent in all field populations but with lower supporting reads in Njombe. It is absent in FUMOZ and has just two supporting reads in FANG. The grey rectangles present on the alignment track of FUMOZ represent normal aligned reads viewed in pairs and separated by a thin line.

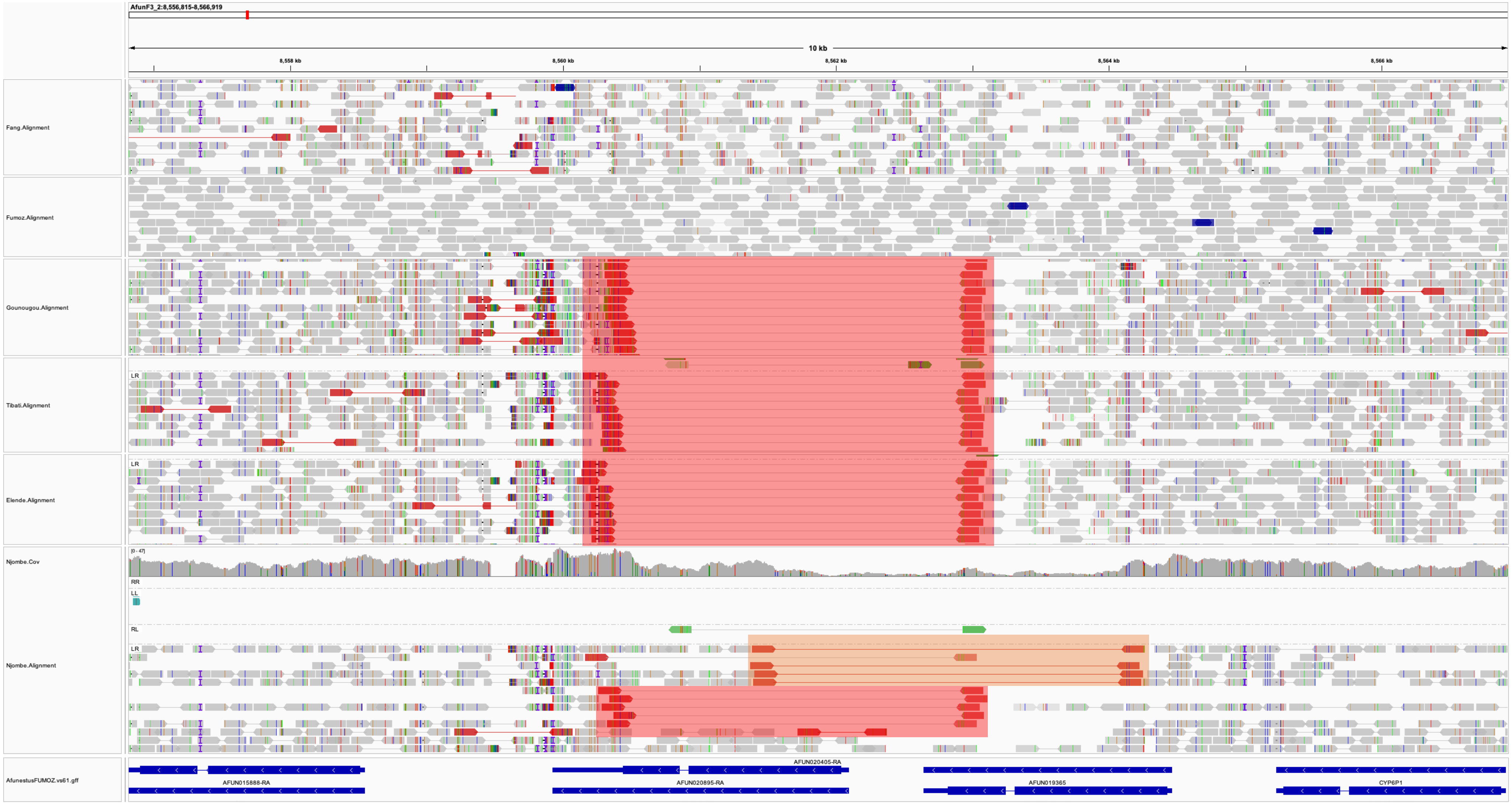


**S6 Fig.** Evidence of a 2.3 kb deletion, spanning partial CYP6P4a and b paralogues, is represented by an unusual large insert size in red rectangles, separated by a thin line and boxed with red boxes. This deletion is more consistent in Gounougou, Tibati, and Elende but with lower supporting reads in Njombe. Conversely, another deletion of 2.5kb was only found in Njombe, shown in an orange box with a drop in coverage depth in this population. All these deletions were absent

in FANG and FUMOZ. The grey rectangles present on the alignment track of FANG and FUMOZ represent normal aligned reads viewed in pairs and separated by a thin line.

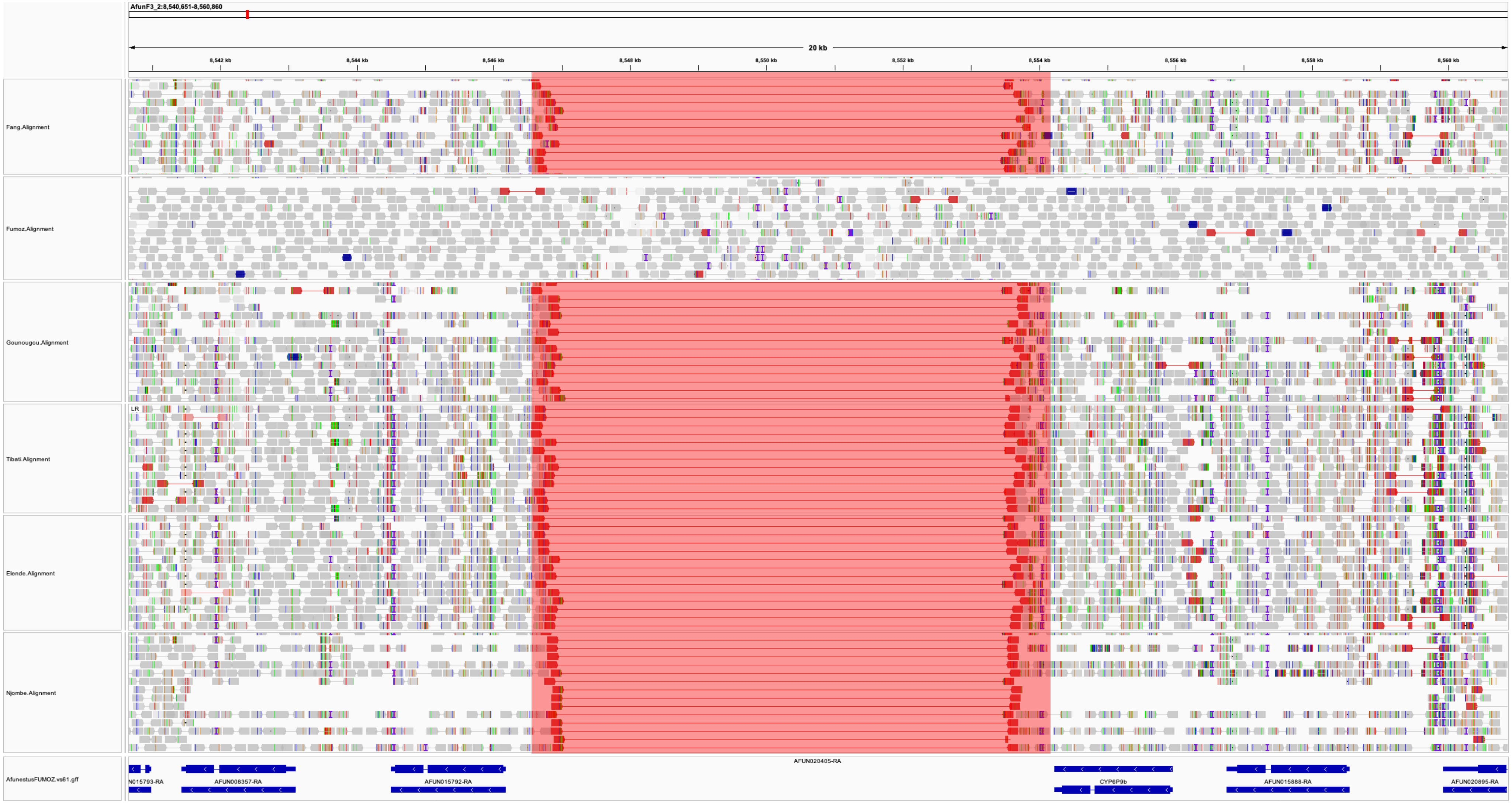


**S7 Fig.** Evidence of a 6.5 kb deletion between CYP6P9a and b (intergenic) paralogues is represented by an unusual large insert size in red rectangle, separated by a thin line and framed in red boxes. This corresponds to an insertion in FUMOZ, which is absent in all our field Cameroon populations, including FANG. The grey rectangles represent normal aligned reads viewed in pairs and separated by a thin line.

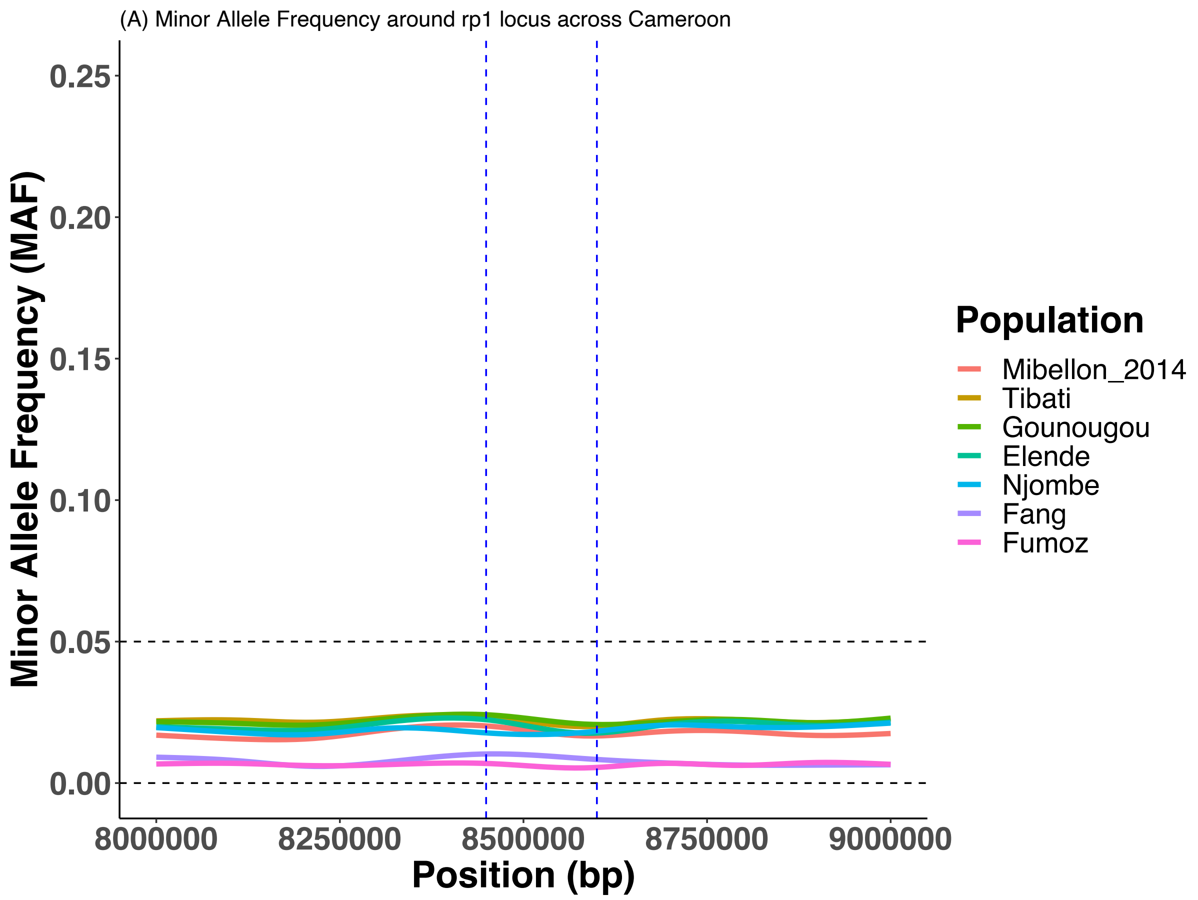

(A) Minor Allele Frequency around rp1 locus across Cameroon
0.25
0.20
0.15
0.10
0.05
0.00
Minor Allele Frequency (MAF)
8000000
8250000
8500000
8750000
9000000
Position (bp)
Population
Mibellon_2014
Tibati
Gounougou
Elende
Njombe
Fang
Fumoz


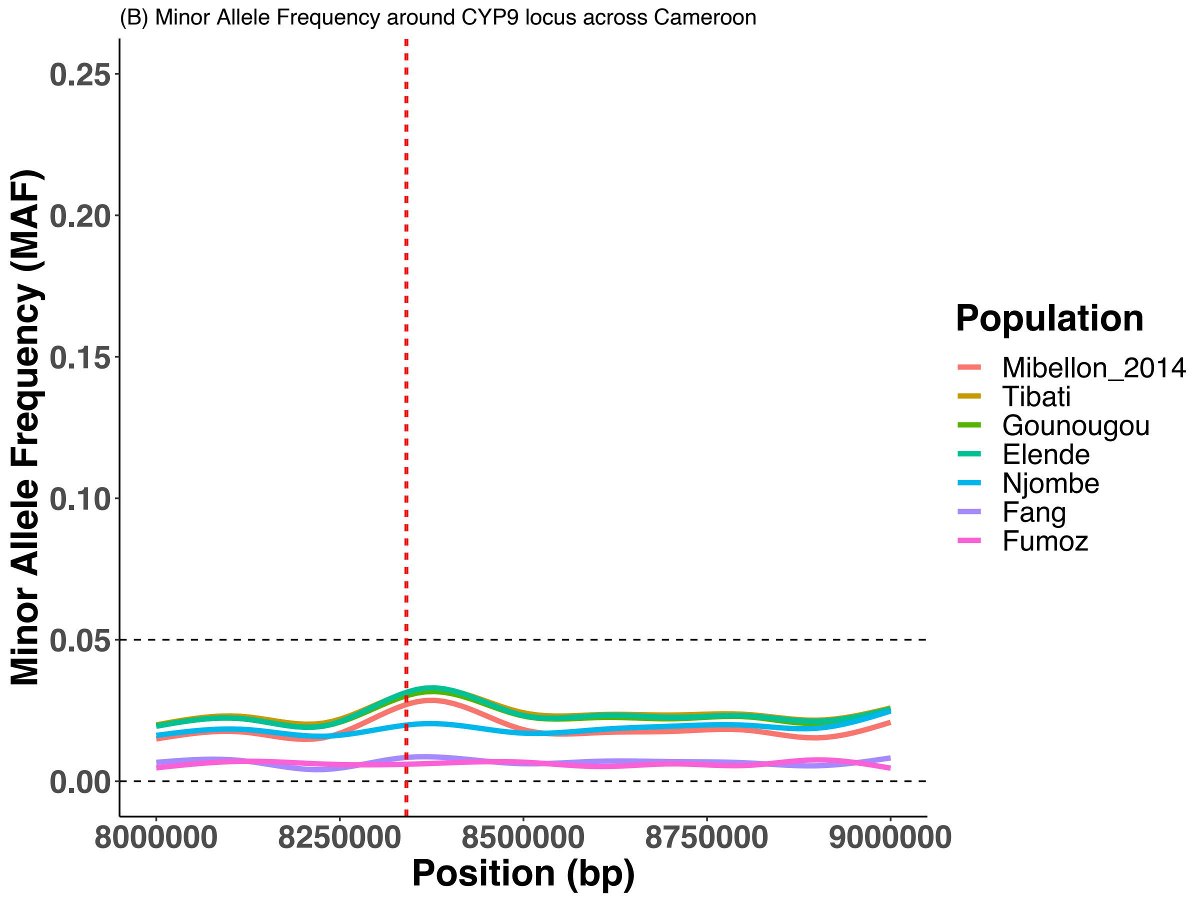

(B) Minor Allele Frequency around CYP9 locus across Cameroon
0.25
0.20
0.15
0.10
0.05
0.00
Minor Allele Frequency (MAF)
8000000
8250000
8500000
8750000
9000000
Position (bp)
Population
Mibellon_2014
Tibati
Gounougou
Elende
Njombe
Fang
Fumoz

**S8 Fig.** Minor allele frequencies spanning the entire rp1 and CYP9K1 loci in *Anopheles funestus* populations across Cameroon.

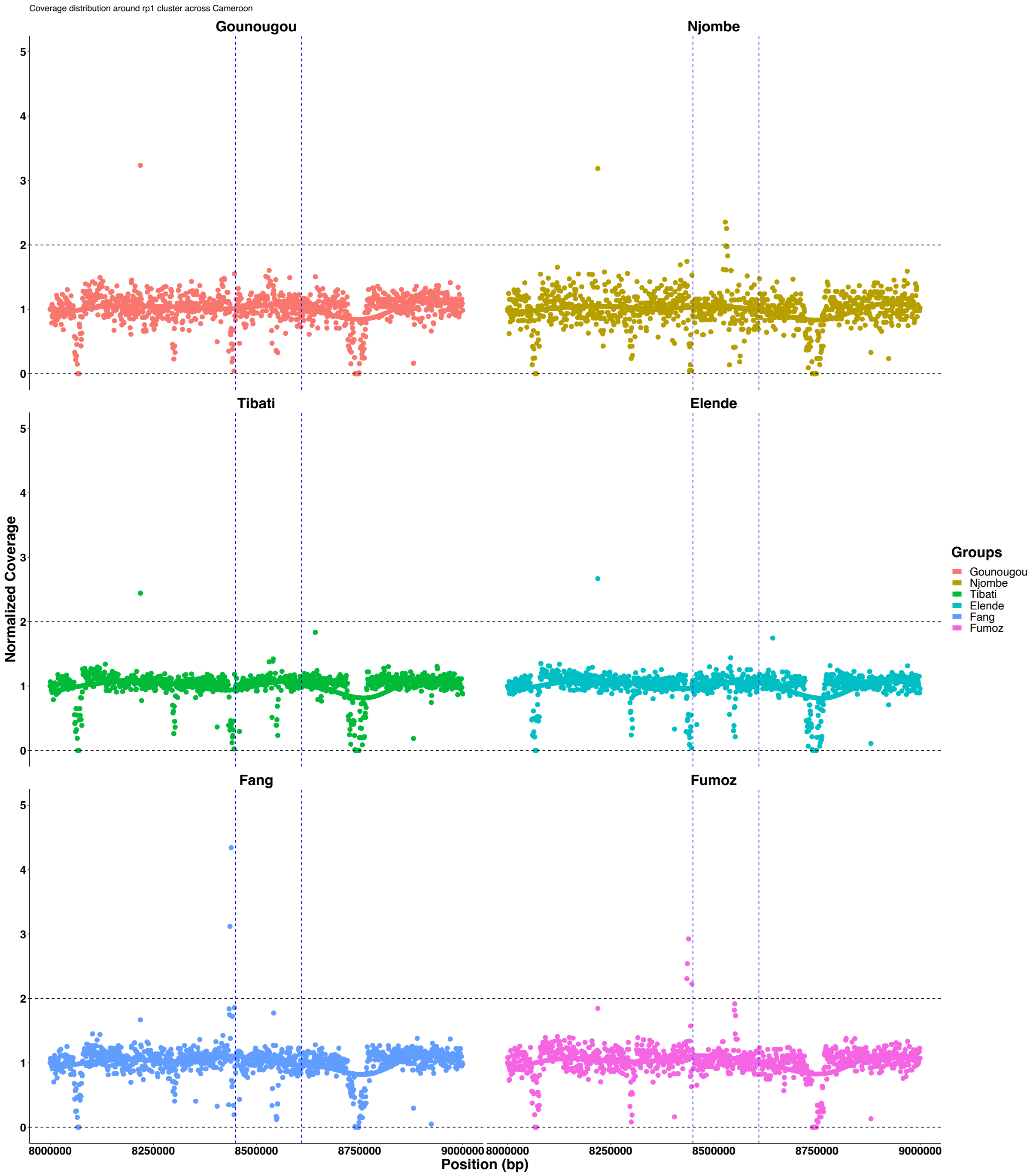


**S9 Fig.** Coverage analyses showing regions potentially affected by CNVs around the QTL rp1 locus in *An. funestus* populations across Cameroon.

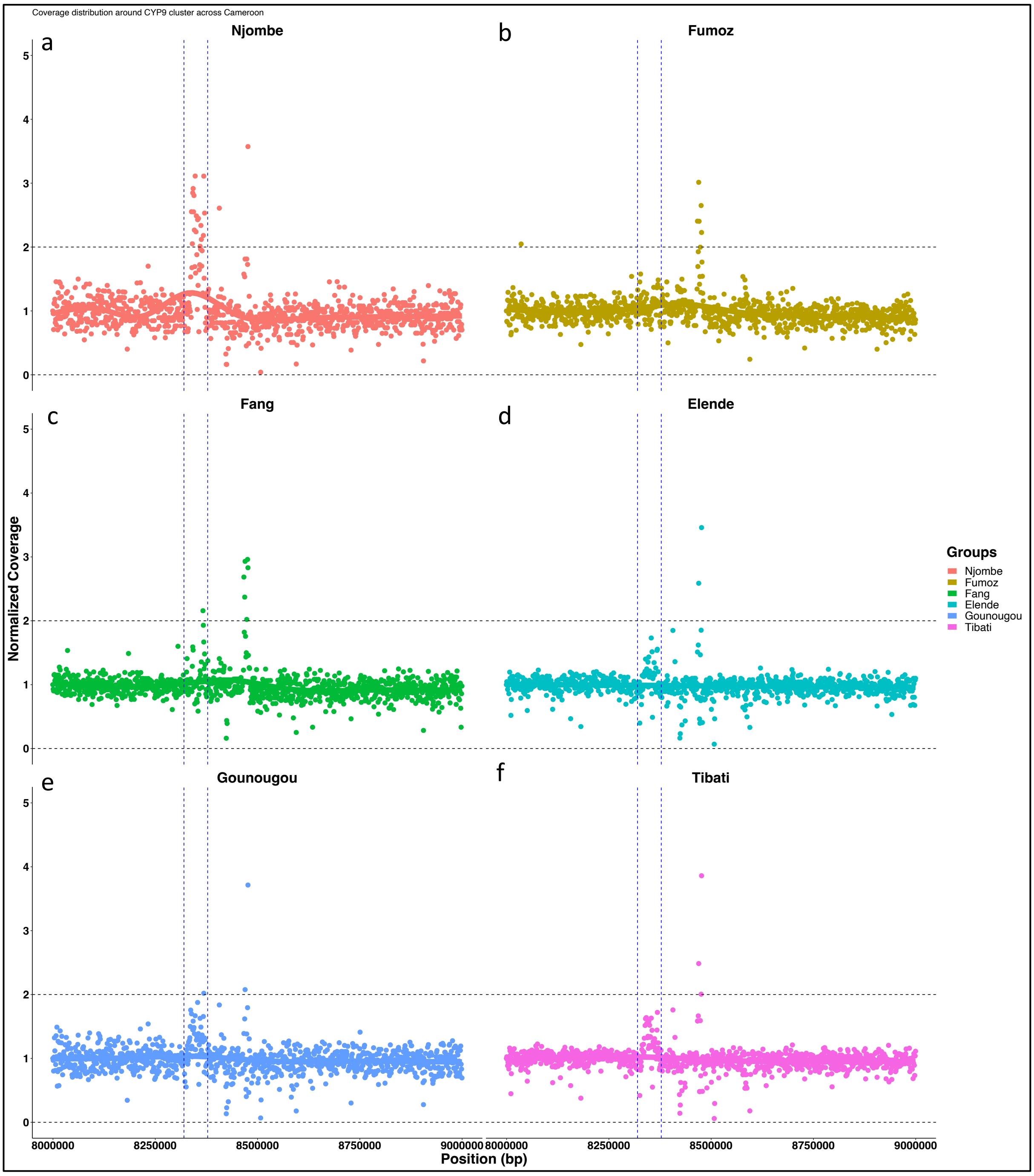


**S10 Fig.** Coverage analyses showing regions potentially affected by CNVs around the CYP9 cluster in An. funestus populations across Cameroon. Each dot represents a coverage window of 1000 bp.
